# Errorful learning of trivia questions and answers: The role of study time

**DOI:** 10.3758/s13421-024-01608-6

**Published:** 2024-07-25

**Authors:** Ewa Butowska-Buczyńska, Maciej Hanczakowski, Katarzyna Zawadzka

**Affiliations:** 1 Department of Cognitive Psychology, Development and Education, SWPS University, Ul. Chodakowska 19/31, 03-815 Warsaw, Poland; 2 Faculty of Psychology and Cognitive Science, Adam Mickiewicz University, Ul. Szamarzewskiego 89AB, 60-568 Poznań, Poland

**Keywords:** Errorful learning, Guessing, Trivia learning

## Abstract

Errorful learning—asking questions and forcing responding even before the correct answers are presented for study—has recently been proposed as a way of maximizing the effectiveness of study. However, much support for the superiority of errorful learning over standard learning via reading comes from studies employing pairs of words as study materials, which remain of little educational relevance. Studies using materials affording richer semantic processing, such as trivia questions and their answers, have shown benefits of errorful learning only when the errorful learning condition is granted additional time for formulating guesses. In the present study, we systematically examined the role of timing when comparing errorful learning and reading strategies applied to study of trivia questions and their answers. In Experiments 1 and 2, we obtained evidence for the superiority of errorful learning over reading when additional time was given to formulate guesses, but this superiority was abolished when the overall time to study was equated between the two learning strategies. We further examined the role of answer familiarity in Experiment 3, showing that incorrect guessing produced no benefit for learning regardless of whether the to-be-learned concepts were familiar or not. In Experiments 4 and 5, no benefits of errorful learning emerged when participants were required to guess responses to two different questions that shared a common set of possible answers. We conclude that the benefits of errorful learning for trivia questions emerge only when guessing gives more time to process target questions.

**Supplementary Information:**

The online version contains supplementary material available at 10.3758/s13421-024-01608-6.

Designing effective learning strategies is an important agenda for researchers interested in memory processes. Much effort has been devoted to outlining strategies resulting in effective learning, with the majority of studies investigating various learning techniques involving retrieval from memory (see Agarwal et al., 2021; Roediger & Karpicke, 2006; Rowland, 2014, for reviews). It has been argued that not only do retrieval attempts enhance memory for the information that is retrieved, but they also potentiate learning of new information that follows retrieval (see Chan et al., 2018; Yang et al., 2018, 2019). A specific case of such new learning augmented by preceding retrieval attempts is referred to as errorful learning. Here, it is argued that being presented with a memory question, attempting to guess the response to this question, and only then seeing the correct answer as feedback, benefits memory for this feedback compared with reading the question together with its answer, with no preceding retrieval attempt (e.g., Kornell et al., 2009; Potts & Shanks, 2014; Yan et al., 2014).

Potential applications of this errorful learning strategy to educational contexts are obvious. If incorrect guessing leads to better memory for feedback, then teachers introducing a novel concept in the classroom should first pose a question concerning this concept and only provide students with the correct response after giving them some time to formulate their guesses. However, one could also argue that such a strategy could incur costs to student learning. For example, having to guess an answer that is unlikely to be known could induce test anxiety in students, or have them question their self-efficacy. What is more, it needs to be noted that having students guess an answer to a question before giving them the correct answer involves an additional cost in the form of extended time necessary for guesses to be formulated. This cost would perhaps be negligible with a single question, but if multiple concepts were introduced in this way, the time spent on guessing could be long enough to prevent the teacher from engaging in other important activities, like answering student questions or providing clarifications. Would guessing still prove superior in supporting memory if compared against conditions assigning this additional time to processing questions and their answers together? Surprisingly little work has been devoted to this issue when considering errorful learning of educationally relevant materials. Thus, the purpose of the present study was to assess the effectiveness of errorful learning as a function of time devoted either to formulating guesses or reading responses to memory questions.

While research on errorful learning has commonly been discussed in terms of its educational applications, the paradigm most often used to investigate errorful learning is far removed from standard classroom situations. In the word-pair learning paradigm developed by Kornell et al. (2009), participants are presented with pairs of weakly related words (e.g., *pond–frog*) to memorize. In the reading condition, participants simply try to learn those pairs for a subsequent memory test. In the errorful learning condition, participants are first presented with the first word as a cue, then asked to guess what the second word could be, before being presented with the whole cue–target pair as feedback. Numerous studies using this paradigm have shown better subsequent cued-recall performance in the errorful learning than in the reading condition (e.g., Bridger & Mecklinger, 2014; Carneiro et al., 2018; Grimaldi & Karpicke, 2012; Huelser & Metcalfe, 2012; Kliegl et al., 2023; Knight et al., 2012; Leggett & Burt, 2021; Pan & Rivers, 2023; Seabrooke et al., 2021; Vaughn & Rawson, 2012; Zawadzka & Hanczakowski, 2019). Studies employing this paradigm were also used to home in on the mechanism that underlies the benefits of errorful learning. The leading account proposed by Grimaldi & Karpicke (2012) states that guessing activates a semantic network of concepts related to the cue. These concepts then facilitate encoding of the target if it also belongs to the common semantic network. This explanation accords well with the lack of guessing benefits when studying semantically unrelated materials, where targets cannot be activated from an unrelated cue (Huelser & Metcalfe, 2012). It also explains the lack of benefits when feedback is presented after a delay (Vaughn & Rawson, 2012), as this semantic activation tends to be rather short-lived, at least when simple study materials are used.

Although the word learning paradigm of Kornell et al. (2009) provides a good opportunity for theoretical progress concerning the mechanisms behind errorful learning benefits—with its simplified materials and highly replicable benefits of guessing—the arbitrary pairings of cues and “correct” responses mean that this paradigm obviously lacks educational relevance. Thus, before recommending errorful learning as an effective learning strategy, it is important to extend findings from this paradigm to other learning materials.

Here, three strands of research need to be mentioned. First, there are studies looking at errorful learning in the context of studying text passages. For example, Richland et al. (2009) showed benefits of committing errors for questions that preceded learning of a science text. However, whenever the initial questions used to elicit errors pertain only to the part of the study material, any effect of such questions can be ascribed to attentional factors. Here errorful learning could have an effect of directing attention toward specific fragments of the text that serve to answer these questions, facilitating their encoding (Carpenter et al., 2018). This attentional effect cannot be easily disentangled from any possible direct effect of errorful learning on memory. That is why in other strands of research on errorful learning it is ensured that the to-be-learned information is not hidden among other, nonrelevant information that needs to be sifted through.

In another strand of research employing materials of direct educational relevance, some studies have looked at whether guessing may augment memory for translations of foreign vocabulary. Results from these studies put a strong limit on the effectiveness of errorful learning, as they found incorrect guessing to lead to *worse* memory compared with reading translations outright (Butowska et al., 2022; Seabrooke et al., 2019). This is not entirely surprising, because guessing for such materials can be compared with guessing when learning unrelated pairs of words. In both cases, cues are not capable of activating targets, either because they are not related to these targets or because this relationship is not established in participants’ semantic memory, and without semantic activation spreading to targets the benefits of errorful learning are unlikely to emerge.

The third line of research uses trivia as learning materials. Here, it is argued that this setup closely resembles the process of learning novel concepts across a variety of domains encountered in educational practice, like biology, history, or geography. Kornell et al. (2009), apart from investigating errorful learning of weakly related pairs of words, adapted the same approach to examining learning of answers to trivia questions and showed better subsequent memory performance when those answers were preceded by guessing. Indeed, a subsequent study by Kornell (2014) showed that errorful learning may be a particularly useful strategy for trivia, even better than for word pairs. This is because the benefits of errorful learning for trivia seem to survive the delay between formulating a guess and the provision of corrective feedback. Kornell demonstrated that those benefits persist across delays of both 6 min and 24 h when trivia are used, while even a short delay is known to eliminate such benefits for word pairs (Vaughn & Rawson, 2012; but see Zawadzka et al., 2023). This robustness of errorful learning benefits obtained for trivia could be due to these materials’ semantic richness, ensuring that the activation arising at the time of guessing is more lasting, facilitating encoding of still activated answers even after a delay.

Before recommending errorful learning as an effective strategy for use in classroom settings, however, one important feature of studies employing word pairs and trivia needs to be highlighted. The studies using weakly related word pairs as study materials commonly equate the duration of a learning trial across errorful learning and reading conditions. Thus, for example, if participants in the errorful learning condition have 5 s to formulate their guess for a particular cue and then they see this cue paired with its associated target for another 5 s, then the duration of the reading trial is a combined 10 s. This equating of time across conditions ensures that any benefit observed in the errorful learning condition cannot be ascribed to longer processing, thus isolating the actual effect of formulating a guess. However, it also means that the errorful learning condition is put at a disadvantage, as participants process the to-be-learned target for a substantially shorter time compared with the reading condition. The fact that the benefits of errorful learning are ubiquitously observed under those equated time conditions testifies to the robustness of this strategy for learning weakly related word pairs. The reading of studies using trivia questions and answers as study materials, however, provides a much less clear picture concerning the effectiveness of errorful learning.

The study by Kornell (2014) demonstrated the benefits of errorful learning for trivia under conditions of delay between guessing and the presentation of feedback. However, in the first experiment documenting this effect (Experiment 2), the time to process questions together with their answers was equated across the errorful learning and reading conditions (8 s), while the additional time devoted to reading questions and formulating guesses in the errorful learning condition (12 s) was not accounted for. This effectively means that although participants’ memory was superior in the errorful learning condition, this benefit came at a cost of spending more than twice the time on learning. Two further experiments reported the same benefits of errorful learning for trivia questions, but these experiments used a self-paced procedure, so it remains unknown how much additional time was spent by participants on formulating guesses in the errorful learning condition. Interestingly, an earlier study by Kornell et al. (2009) included two experiments examining errorful learning of fictional trivia statements, with Experiment 1 equating the time to read answers in the errorful learning and reading conditions (5 s) but not accounting for the time to formulate guesses (8 s) and Experiment 2 equating the duration of the whole learning trial by extending reading trials (13 s). With this design, the benefits of errorful learning emerged in Experiment 1, but not in Experiment 2 (see also Krogulska et al., 2023, for a more recent example of no benefits of errorful learning for trivia with trial time equated).

Overall, thus, the benefits of errorful learning of trivia seem readily observed when participants have additional time to formulate their guesses in the errorful learning condition, but the status of this effect when learning duration is equated across errorful learning and reading conditions remains uncertain. Not only does this stand in contrast with studies using pairs of words as study materials, where clear benefits of errorful learning are found even when duration of the whole study episode is equated across learning conditions, but it also undermines the potential usefulness of the guessing strategy in many educational contexts. Does it make sense to require students to guess at answers they cannot possibly know when the same memory effect can be achieved simply by extending study time? Is there really an additional benefit resulting from engaging in futile retrieval attempts?

Here, we examined this issue closely in a series of five experiments involving learning answers to trivia questions either via errorful learning, requiring guessing at correct answers, or reading answers presented outright with their questions. In this examination, we employed larger samples of participants than the ones tested in the original study of Kornell et al. (2009) to ensure adequate power to detect any benefits accruing from errorful learning. We used true (rather than fictional) trivia questions of the sort used in the study by Kornell (2014) to ensure educational relevance of the examined learning strategy. In Experiment 1, we examined the role of trial duration across the errorful learning and reading conditions in one experimental design, contrasting directly trials equating the whole duration of the study episode and trials equating the duration of answer processing. In Experiment 2, we focused more directly on trials that equated the whole duration of the study episode. In Experiment 3, we attempted to isolate the effects of errorful learning with equated processing time for questions with familiar and unfamiliar answers. In Experiments 4 and 5, we assessed the role of semantic activation in errorful learning of trivia by requiring participants to guess an answer to a related question before learning the answer to the target question.

To foreshadow, we failed to document any benefits of errorful learning when the total time devoted to study was equated with the time to process the question and its answer together in the reading condition. Those benefits emerged clearly only when participants in the errorful learning condition had more time to process exactly the same question for which the correct answer was later delivered. This indicates that the benefits of errorful learning when trivia are studied are likely to reflect extended processing of a question rather than any beneficial effects of semantic activation caused specifically by incorrect guessing.

## Experiment 1

Experiment 1 assessed the role of total trial duration in driving the performance differences between errorful learning and reading strategies. Participants had to study various difficult trivia questions with their correct answers. In the errorful learning condition, they were asked to type in within 8 s their best guess as to what the answer to a given question could be, after which they were presented with corrective feedback in the form of the right answer for 5 s. In the short reading condition, the answer was presented outright, and the duration of the learning trial was equated with the duration of feedback presentation in the errorful learning condition (5 s). In the long reading condition, the answer was also presented together with the question, but the duration of the learning trial was equated with the duration of the whole trial from the errorful learning condition (13 s). The learning phase was followed by a final cued-recall test, in which participants were presented with the studied trivia questions and asked to provide the correct response. If errorful learning leads to better encoding of answers to trivia questions, we would expect performance in the errorful learning condition to be better than in both the short and long reading conditions. If, however, the time to process questions is vital, then we would expect performance in the errorful learning condition to be no different from performance in the long reading condition, with both outstripping performance in the short reading condition.

### Method

#### Participants

The sample size was based on the previous investigation of errorful learning with trivia questions and answers by Kornell (2014, Experiment 2), where the memory benefits of errorful learning were obtained with *d* = 0.58. Thus, to achieve power of 0.95 to detect a difference between the errorful learning and short reading conditions, 41 participants would have to be tested. Forty-three participants recruited via the Prolific website (age range 24–68 years, *M* = 38.4, *SD* = 11.9) participated in Experiment 1 in exchange for monetary compensation. Three participants were above the age of 60.^1^ Out of all tested participants, three were excluded due to close-to-zero accuracy on the final test, which gave a total of 40 participants. All participants were native English speakers. The majority of participants were born in the United Kingdom (20 participants) or the United States (18 participants), and there were also single participants born in Poland and South Africa. The study was approved by the Department of Psychology Ethics Committee at the SWPS University.

#### Materials and design

**Fig. 1:**
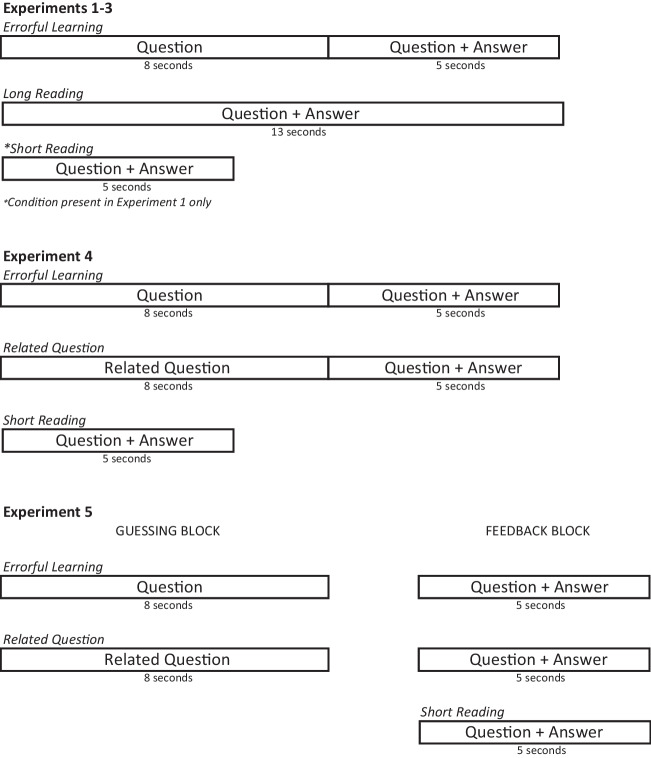
Study trial timings across Experiments 1–5

Forty-eight questions taken from the study by Kornell (2014) were used as study materials (e.g., *What is the world’s tallest grass*? Bamboo), and three additional new questions were used for practice. The question list was divided in three, with each third assigned to a different learning condition. Figure 1 shows the study trial timings which defined the conditions. Following Kornell et al. (2009), in the long reading condition, the question and the correct response were presented together for 13 s. In the short reading condition, the question was presented together with the correct response for 5 s. In the errorful learning condition, the question appeared first for 8 s, and after that the correct response appeared beneath the question for 5 s. Although we were aware of possible negative effects of time pressure for learning, study time duration was set by the experimenter rather than self-regulated across all experiments reported here. This is because the aim of the study was to provide a comprehensive test of the efficacy of errorful learning compared with reading specifically under equated time conditions. The study phase was followed by a cued-recall test, in which participants were presented with the questions as cues and had to type in the response. Thus, the study had three learning conditions (long reading vs. short reading vs. errorful learning) manipulated within participants. The assignment of questions to conditions was counterbalanced across participants, and the order of question presentation at study and test was randomized anew for each phase and each participant.

#### Procedure

Participants were tested individually online. They were instructed that their task would be to learn answers to trivia questions, which would be encountered in three different learning scenarios. For some trials, the question would appear first and they would be required to type in their best guess as to what the answer might be within 8 s, before being provided with the correct answer for 5 s. For some other trials, the correct response would be presented outright with the question either for 13 or 5 s. Participants were also instructed not to use any external resources and rely solely on their memory while doing the experiment.

After these initial instructions, participants engaged in a short training phase consisting of studying three trivia questions in the three learning conditions. Then, they completed the study phase followed by a memory test that presented the trivia questions from the study phase as cues and required typing in the correct responses. The time for producing a response in the final memory test was not limited.

### Results and discussion

During the learning phase, 53 questions were correctly answered in the errorful learning condition, which constituted 8.3% of all trials. The average number of correctly answered items per participant was thus 1.33 (*SD* = 1.14). Since the focus of the study was on the effects of incorrect guessing on learning, these trials were removed from the analyses of final cued-recall performance here and in all subsequent experiments, as is common practice in studies on errorful learning (Kornell et al., 2009). It is worth bearing in mind that such removal skews the recall results against any benefits in the errorful learning condition. Questions for which correct responses are produced during learning are those for which participants know the correct answer and thus would be highly likely to be answered correctly in the final test. Such questions are not removed from either of the reading conditions, contributing to correct responding in these conditions. To make sure that removing these questions did not alter the pattern of results, here, and for all subsequent experiments, we also performed analyses on the full dataset, which are reported in footnotes.

Table 1 presents cued-recall performance across learning conditions. A one-way analysis of variance (ANOVA) on the proportion of correct responses on the final test showed a significant difference between the learning conditions, *F*(2, 78) = 5.62, *p* = .005, η_p_^2^ = .13. This was followed up with a series of paired-sample Bonferroni-corrected *t* tests to compare performance across the conditions. Performance in the errorful learning and long reading conditions was nearly identical and not significantly different, *t*(39) = 0.013, *p* = 1.00, *d* < 0.01. Performance in the short reading condition was, on the other hand, significantly lower than in the errorful learning, *t*(39) = 2.74, *p* = .028, *d* = 0.43, and long reading conditions, *t*(39) = 2.72, *p* = .029, *d* = 0.43. The same pattern of results was found in this and all further experiments when the analyses were performed on the full dataset, without excluding questions for which correct answers were provided during the guessing stage.^2^

**Table 1:** Cued recall performance as a function of learning condition across Experiments 1–5

	Learning condition			
Experiment	Errorful learning	Long reading	Short reading	Related question
Experiment 1	.83 (.03)	.83 (.03)	.77 (.04)	–
Experiment 2	.79 (.02)	.79 (.02)	–	–
Experiment 3				
Familiar targets	.89 (.03)	.88 (.03)	–	–
Unfamiliar targets	.33 (.04)	.39 (.04)	–	–
Experiment 4	.63 (.02)	–	.57 (.02)	.56 (.02)
Experiment 5	.61 (.02)	–	.57 (.03)	.56 (.02)

Standard errors of the mean are given in parentheses

Even though our initial plan was to use frequentist statistics for this research project, the results of the ANOVA demonstrated that this approach was insufficient, as it did not allow for establishing whether the lack of a significant difference between our two conditions of interest reflected a true lack of a difference or just a false negative. Thus, for this experiment, as well as for subsequent ones, we also report Bayesian statistics. A Bayesian repeated-measures ANOVA conducted in JASP (JASP Team, 2023) with default priors yielded moderate evidence for the effect of learning condition, BF₁₀ = 6.73. Importantly, a Bayesian *t* test comparing performance in the errorful learning and long reading conditions yielded moderate evidence in support of the null hypothesis, BF₀₁ = 5.89, which is suggestive of the lack of a significant difference between the conditions reflecting an actual lack of an effect. Further *t* tests yielded moderate evidence for a difference between the errorful learning and short reading conditions, BF₁₀ = 4.34, as well as between the long reading and short reading conditions, BF₁₀ = 4.19.

The results of the first experiment clearly show the influence of total study time on the effectiveness of learning, while at the same time casting a shadow on the role of errorful learning in supporting memory. When the total study time was equated, there was no difference in resulting memory performance from incorrect guessing and reading. As such, the present results replicate those reported by Kornell et al. (2009) in their investigation of guessing answers to fictional trivia questions (see also Krogulska et al., 2023). At the same time, both conditions using longer exposure to trivia questions produced better learning compared with the short reading condition, where the time to process the questions was more limited. This also remains consistent with previous studies where such benefits emerged (Kornell, 2014; Kornell et al., 2009). Still, before concluding that there is at best a limited benefit from engaging in errorful learning when the total time to process question is held constant, we decided to conduct one more experiment, employing the Bayesian approach to data analysis to quantify the evidence for a null effect, to ensure the robustness of the null difference between the errorful learning and long reading conditions.

## Experiment 2

Experiment 2 included only two learning conditions—the errorful learning condition and the long reading condition (see Fig. 1). The sole aim of this experiment was to provide stronger evidence against errorful learning improving memory for answers to trivia questions when the time to process questions is equated with the time available in the reading condition.

### Method

#### Participants

Seventy-three participants recruited via the Prolific website (age range: 20–75 years, *M* = 36.8, *SD* = 12.24) participated in Experiment 2 in exchange for monetary compensation. Four participants were above the age of 60. All participants were native English speakers. Two participants were excluded due to substantial (more than 0.50) accuracy when providing initial guesses while learning the questions, which gave a total of 71 participants. The majority of participants were born in the United Kingdom (47 participants), the United States (10 participants), and South Africa (seven participants), and there were single participants born in Australia, Canada, Czechia, Iran, Ireland, Pakistan, and Romania. We aimed to recruit 70 participants and then assess the Bayes factor, assuming that we would continue testing if it remained uninformative (i.e., below 3 in support for either the null or the alternative hypotheses).

#### Materials, design, and procedure

Materials, design, and procedure were identical to those of Experiment 1, except for one difference. Namely, we excluded the short reading condition, which gave us a larger number of items to be studied and analyzed in each learning condition compared with Experiment 1 (24 instead of 16).

### Results and discussion

During the learning phase, 112 targets were correctly guessed. This resulted in 6.5% of trials with a correct guess being removed from subsequent analyses. The average number of correctly answered items per subject was thus 1.6 (*SD* = 1.54).

As shown in Table 1, performance in the two learning conditions was nearly identical and not significantly different, *t*(70) = 0.14, *p* = .89, *d* = 0.02.^3^ A Bayesian paired-samples *t* test conducted in JASP (JASP Team, 2023) with its default priors yielded moderate evidence in support of the null hypothesis, BF₀₁ = 7.59. Thus, the results of Experiment 2 are consistent with the results of Experiment 1 in failing to show any effect of learning condition when the time devoted to processing questions is equated across conditions. Whether participants formulated incorrect guesses or simply spent this time reading questions and answers together had no bearing on the effectiveness of learning. Once again, these results align with previous research on guessing answers to fictional trivia questions (Kornell et al., 2009; Krogulska et al., 2023) and cast shadow on the effectiveness of errorful learning as applied to materials other than weakly related pairs of words.

The obvious question at this point is why an effect readily observed with materials such as weakly related word pairs is absent when trivia questions and answers are studied. It is perhaps worth reiterating here that there are other situations in which errorful learning fails to support memory, and which involve learning the meanings of unfamiliar words, such as, for example, words in a foreign language (Butowska et al., 2022; Seabrooke et al., 2019). Indeed, in these situations, errorful learning has been shown to result in poorer learning of associations between foreign words and their translations than simply reading those two together. The crucial issue seems to be the transparency of the relationship between cues and their targets. When this relationship is transparent, as it is virtually always the case for weakly related pairs of words, errorful learning is beneficial, presumably because transparent semantic relationships allow for spreading of semantic activation caused by guessing. However, when this relationship is opaque, as necessarily occurs for all cases where one of the to-be-associated words is not known, semantic activation does not spread and errorful learning may even be detrimental when incorrect guesses interfere with learning or retrieval of correct answers.

When this transparency-based distinction is applied to trivia questions and answers, one could speculate that these materials may afford a mix of these two cases, with errorful learning facilitating learning of answers already perceived to be related to their questions but impeding learning of answers that are completely novel. A net result of this could be an overall lack of memory benefits of guessing answers to trivia questions. In Experiment 3, we attempted to disentangle these two situations by examining the effects of errorful learning separately in cases when the answers to a question were familiar concepts, presumably already related to their questions, and when these answers were novel concepts.

## Experiment 3

In Experiment 3, we again assessed whether errorful learning improves memory for trivia questions and answers when the time of learning is equated with the control condition of reading. Here, we introduced a new factor—target familiarity. We hypothesized that while errorful learning may improve memory for trivia questions for which answers are familiar, it may impair memory for cases in which those answers are unfamiliar. That errorful learning may harm performance specifically when studying unfamiliar novel words has been shown in previous research which evidenced an errorful learning disadvantage when forming associations between novel rare or foreign words and their translations (Butowska et al., 2022; Seabrooke et al., 2019).

### Method

#### Participants

Forty-one participants recruited via Prolific (age range: 21–85 years, *M* = 38.5, *SD* = 14.57) participated in Experiment 3 in exchange for monetary compensation. Four participants were above the age of 60. The sample size we aimed for was the same as in Experiment 1. All participants were native English speakers. To make sure that the targets were (un)familiar to a similar extent to all participants, we restricted the sample to people living in the United States. Thus, in Experiment 3, all participants were current U.S. residents, and three of them were born outside the United States (Canada, Barbados, Russia).

#### Materials, design, and procedure

For the purpose of Experiment 3, we created a new set of learning materials consisting of 24 questions with familiar answers (e.g., *What was the third name of Queen Elisabeth II*? Mary) and 24 questions with unfamiliar answers (e.g., *What is the longest river in Spain*? Ebro). Some of the questions were identical to those used in Experiments 1 and 2, while some were novel, as in Experiments 1 and 2 we did not control for the familiarity of responses. For the purpose of this study, we developed a procedure aimed at assessing the familiarity of the target answers. Participants recruited via Prolific were presented with 48 targets (half of them presumed to be familiar and half unfamiliar), without their respective questions, and were asked to rate how familiar those targets seemed to them on a scale from 1 (*very unfamiliar*) to 5 (*very familiar*). We first recruited 20 participants, and from their responses we calculated a mean familiarity score for each target and excluded those with scores between 2 and 4. On this basis, we excluded and replaced 13 items. We then recruited another 20 participants to rate the familiarity of each target from the full set. This time all targets met our criteria. In this way, we ended up with a set of 24 unfamiliar (familiarity ratings between 1 and 2, *M* = 1.34) and 24 familiar targets (familiarity ratings between 4 and 5, *M* = 4.81) to be used as study materials.

The design of the experiment included two factors: learning condition (errorful learning vs. long reading) and target familiarity (high vs. low), with both variables manipulated within participants. The assignment of questions to the two learning conditions was counterbalanced across participants, and the order of their presentation for study and for test was randomized anew for each participant and each phase of the procedure. The procedure remained the same as in Experiment 2, with the total duration of a learning trial equated across errorful learning and long reading conditions (13 s in the long reading condition, and 8 s for guessing plus 5 s for feedback in the errorful learning condition; see Fig. 1).

### Results and discussion

During the learning phase, 49 targets (41 familiar and eight unfamiliar) were correctly provided as answers. This resulted in an average of 4.9% (8.3% for the familiar and 1.6% for the unfamiliar condition) trials with a correct answer, which were removed from subsequent analyses. The average number of correctly answered items per participant was 1.2 (*SD* = 1.25).

We analyzed cued-recall performance (see Table 1 for descriptive statistics) with a repeated-measures ANOVA, with learning condition (errorful learning vs. long reading) and target familiarity (high vs. low) as factors. This yielded a significant main effect of target familiarity, *F*(1, 40) = 209.68, *p* < .001, η_p_^2^ = .84. Overall, questions with familiar answers were answered correctly more often (*M* = 0.89, *SD* = 0.17) than questions with unfamiliar answers (*M* = 0.36, *SD* = 0.27). There was no significant main effect of learning condition, *F*(1, 40) = 2.62, *p* = .11, η_p_^2^ = .061. Also, the interaction was not significant, *F*(1, 40) = 2.98, *p* = .09, η_p_^2^ = .07. However, given our specific predictions formulated for the present experiment (i.e., that errorful learning should aid associating questions with familiar, but not with unfamiliar responses), we performed Bonferroni-corrected planned comparisons of the effectiveness of learning strategy separately for questions with familiar and unfamiliar answers. For familiar answers, there was no significant difference between the learning conditions, *t*(40) = 0.22, *p* = 1.00, *d* = 0.03. For unfamiliar answers, long reading was numerically better in supporting subsequent memory than errorful learning. This difference, however, did not reach significance, *t*(40) = 2.37, *p* = .12, *d* = 0.37.^4^

A Bayesian repeated-measures ANOVA yielded extreme evidence in support of the effect of familiarity, BF_incl_ = 3.743e + 38, and moderate evidence against the main effect of learning condition: BF_excl_ = 3.56. The evidence against the interaction was, however, only anecdotal, BF_excl_ = 2.06. Bayesian paired-samples *t* tests comparing errorful learning with long reading when studying familiar responses yielded moderate evidence in favor of the null hypothesis, BF₀₁ = 5.76, and for unfamiliar responses the evidence was inconclusive, BF₁₀ = 1.39.

In short, we again failed to observe the benefits of errorful learning over reading when learning trivia under conditions of equated total learning time. As could be expected, the familiarity of answers to trivia questions had a strong effect on learning—it was easier to learn familiar rather than unfamiliar answers. However, even for familiar answers—where questions and their answers should have a preexisting semantic relationship affording the way for semantic activation to spread when guessing—errorful learning failed to benefit memory. For unfamiliar answers, a trend towards impairment in memory due to errorful learning was present, albeit not significant, which would remain consistent with studies showing a similar impairment when learning materials consist of foreign vocabulary translations (Butowska et al., 2022; Seabrooke et al., 2019).

All experiments presented so far seem to question the effectiveness of errorful learning as a strategy of mastering educationally relevant materials. Once again, and notwithstanding previous studies controlling study time and pointing to a similar conclusion (Kornell et al., 2009; Krogulska et al., 2023), this is surprising inasmuch as the current null results stand in contrast to results of studies employing weakly related word pairs as learning materials (e.g., Grimaldi & Karpicke, 2012). If spreading semantic activation resulting from guessing does support learning of such pairs of words, then why does it seem not to work for trivia questions? In Experiment 3 we assessed—but failed to confirm—whether the problem may lie on the side of the errorful learning condition, where a mixture of familiar and unfamiliar answers could undermine the effectiveness of incorrect guessing. But another possibility is that the problem lies on the side of the reading condition. It could be argued that trivia questions offer such semantically rich encoding opportunities that when they are processed for a substantially long time, semantic activation reaches the threshold of activation caused by incorrect guessing. In other words, under long encoding conditions—for which the errorful learning strategy does not supersede the reading strategy—semantic activation may plateau at the same level for both strategies. But when trivia questions are read for a relatively short time, activation has no time to accrue, resulting in reduced effectiveness of encoding compared with the errorful learning strategy. We aimed to assess this explanation in the next two experiments, where we attempted to de-confound the effects of guessing and learning time by requiring participants to guess the answer not to a question presented for learning, but to a closely related question that should nevertheless activate a common semantic network.

## Experiment 4

In Experiment 4, we aimed to assess the effects of guessing an answer to a related question for subsequent learning of an answer to a target question. Thus, for example, participants could first be asked about the *tallest* U.S. president, and after formulating their guess they would be presented with a question and the correct answer concerning the *shortest* U.S. president. In this case, guessing an answer to the initial question would presumably activate a semantic network common to both the initial and the target question, as their answers are related. This activation should in turn facilitate learning of the correct answer to the target question. It can be argued that such a *related question condition* which requires participants to answer two questions rather than just one generates more interference compared with the standard guessing condition. On the other hand, however, the related questions were designed specifically to activate the same response set as the following target questions, and hence the possible negative effects of interference should be outweighed by the positive effect of semantic activation, as suggested by Grimaldi and Karpicke (2012) for weakly related word pairs.

The effects of such a learning strategy were compared both with the standard condition of errorful learning that requires guessing an answer to the target question itself and with the short reading condition. If semantic activation spreading before the target question is presented increases the effectiveness of learning, then we would expect the related question and errorful learning conditions to result in similar memory performance. At the same time, both conditions should result in better learning than the short reading condition, where semantic activation should not have enough time to develop. However, it may also be the case that the previously observed differences across the errorful learning and short reading conditions reflect merely the difference in the time devoted to processing the target question, as an errorful learning trial provides eight additional seconds for studying the target question compared with a short reading trial. If the study time difference is the main factor contributing to the errorful learning advantage over the short reading condition, then we would expect the related question condition to result in learning being similarly effective as in the short reading condition. This is because the related question condition presents the target question together with its response for as long as the short reading condition, and the additional 8-s long guessing attempt includes a different question and hence does not give an opportunity for additional processing of the target question.

### Method

#### Participants

Because our design now included a new condition, where performance could in principle lie somewhere between the errorful learning and short reading conditions, we aimed to test a larger sample of participants to ensure sufficient power to detect smaller differences across conditions. To obtain power of 0.95 to detect an effect of *d* = 0.40, at least 84 participants would have to be tested. We thus recruited 90 English-speaking participants via the Prolific website (age range: 20–75 years, *M* = 38.5, *SD* = 12.85), who participated in Experiment 4 in exchange for monetary compensation. All participants were current U.S. residents, and six of them were born outside the United States (Canada, India, Korea, Peru, Nigeria, Taiwan). Five participants were above the age of 60.

#### Materials, design, and procedure

In order to match the requirements of the novel guessing condition, we created a new set of 96 trivia questions and answers. In order to create those questions, the first author compiled a list of questions from various Internet sources that were linked by the category to which their targets belonged (e.g., star constellations, female Nobel prize winners, US states, etc.). The list was then reviewed by the remaining authors who chose the final list of 48 question pairs judged as most likely to hint at related concepts and thus activate the same broad set of possible responses. One question was always used as a target question for the learning task, while the other was used in the related question condition as a primer preceding its related target question (e.g., *Which flower is the national symbol of Scotland*? Thistle, followed by *Which flower is the national symbol of England*? Rose). Further information regarding these questions, as well as additional analyses, can be found in the Appendix.

The design of Experiment 4 included three learning conditions: standard errorful learning and short reading conditions, which were identical in structure to those used in Experiment 1, and the novel related question condition, which involved a guessing attempt (8 s) to a related question, followed by an immediate presentation of the target question together with its answer (5 s). Thus, the overall duration of the related question trial was equated with the standard guessing condition (13 s), while the time to process the target question and its answer was equated with the short reading condition (5 s; see Fig. 1).

The procedure of Experiment 4 was akin to previous experiments, with one exception. Participants were warned that when asked to formulate their guesses during study, they would sometimes be presented not with the corrective feedback for this question but rather with a related question and its answer, which they should learn in preparation for the final memory test.

### Results and discussion

During the learning phase, 73 answers to the target questions were correctly guessed in the errorful learning condition, which resulted in 5% of trials being removed from subsequent analyses. The average number of correctly answered items per subject was 0.81 (*SD* = 1.22). Also, 209 responses to the related questions were correct, constituting 14.5% of trials. The reason why related questions were easier to answer compared with target questions was that they were constructed specifically to activate a relevant semantic network; in order to do so, they had to refer to concepts familiar to participants. Since the related questions were not tested themselves, these trials were not removed from the analysis.

A one-way ANOVA revealed significant differences in final test performance between the learning conditions, *F*(2, 178) = 8.18, *p* < .001, η_p_^2^ = .08 (see Table 1 for descriptive statistics).^5^ To unpick these differences, we performed a series of Bonferroni-corrected paired-sample *t* tests. Performance in the errorful learning condition was significantly higher than in the related question condition, *t*(89) = 3.63, *p* = .001, *d* = 0.38, and in the short reading condition, *t*(89) = 3.10, *p* = .008, *d* = 0.33. There was no significant difference between the short reading and related question condition, *t*(89) = 0.73, *p* = 1.00, *d* = 0.08.^6^

A Bayesian repeated-measures ANOVA yielded strong evidence for the impact of learning condition on performance, BF₁₀ = 50.49. Bayesian paired-samples *t* tests shed further light on the findings from frequentist *t* tests, showing strong evidence for an advantage of standard errorful learning over answering a related question first, BF₁₀ = 45.36, and moderate evidence for an advantage of errorful learning over short reading, BF₁₀ = 9.81. A comparison of the related question and short reading conditions yielded moderate evidence in support of the null hypothesis, BF₀₁ = 6.61.

The current results once again replicated the standard guessing benefit over the short reading condition, also documented here in Experiment 1 and in previous studies on errorful learning using trivia as study materials (Kornell, 2014; Kornell et al., 2009). Crucially, though, the results were also quite clear in showing no memory benefit of the related question condition over the short reading condition. These results suggest that formulating guesses allows for extended processing of a question rather than for semantic activation which further translates into a memory benefit. If guessing were to activate a semantic network common to the guess and the target answer, facilitating encoding of activated information, we would also expect guessing an answer to a related question to benefit learning of the subsequent target answer. That such benefits failed to emerge suggests that the benefits observed in the standard errorful learning condition may be merely due to additional time to process the exact question for which an answer needs then to be learned. Of course, it is possible to argue that our related questions were not related enough, often failing to activate relevant knowledge. It is worth noting, however, that the results revealed no trend whatsoever for the benefits of answering related questions compared with the short reading condition, with means in the wrong direction. It would thus be necessary to argue that virtually none of our related questions activated relevant knowledge, which seems to us not plausible.

Still, an alternative account for the present failure to find learning benefits in the related question condition needs to be considered. In this condition, we first asked participants to generate a response to a related question, only to immediately change the question to a similar one and ask participants to memorize it together with its response. This design generates potential challenges for participants, as they are forced to constantly switch between retrieval and encoding of various study materials. Such switches between retrieval and encoding may engender a cost to the effectiveness of the learning process. Finn & Roediger (2013) demonstrated that retrieving associations between studied faces and names hinders the incorporation of new associative information (profession) compared with restudying the same association. Also, Davis et al. (2017) showed that interpolating retrieval of old material with studying new material impaired new learning proportionally to the frequency of switching between retrieval and learning. Applied to the present paradigm, thus, switching between retrieval of potential answers to related questions and encoding of target questions and answers could generate switch costs, potentially masking the beneficial effect of semantic activation resulting from guessing required for related questions. To assess this explanation of the apparent lack of learning benefits in the related question condition, in Experiment 5 we separated the phases of guessing and learning target questions together with their answers, in this way avoiding any switch costs in the related question condition.

## Experiment 5

The main aim of Experiment 5 was to assess the effectiveness of learning trivia questions and their answers when preceded by a guessing attempt to a related question. In this experiment, guesses for all questions—including guesses to related questions and guesses to target questions in the standard errorful learning condition—were formulated in a separate phase of the experiment, before the proper study session in which target questions were presented together with their answers. The effectiveness of learning in the related question and errorful learning conditions was once again compared against the baseline of the short reading condition. Kornell (2014) showed that the benefits of errorful learning for trivia still emerge in a blocked design, with a delay between guessing and feedback presentation. Thus, we again expected learning to be more effective in the errorful learning condition than in the short reading condition. If this benefit stems from long-lasting semantic activation caused by guessing, then we would expect a similar benefit to emerge in the related question condition. If, however, the benefits in the errorful condition reflect merely the increased time to process the target question itself, we would again expect the related question condition to result in no better learning than the short reading condition.

### Method

#### Participants

Ninety English-speaking participants located in the U.S. recruited via the Prolific website (age range: 19–64 years, *M* = 45, *SD* = 13.86) participated in Experiment 5 in exchange for monetary compensation. The sample size was the same as in Experiment 4. Fourteen participants were above the age of 60. All participants were current U.S. residents, and nine participants were born outside the United States (two in Trinidad and Tobago, one each in Australia, Canada, Ireland, Korea, Macedonia, Slovenia, Venezuela). One person was excluded due to close-to-zero final accuracy, which resulted in a total of 89 participants.

#### Materials, design, and procedure

The materials and design were identical to those from Experiment 4. The procedure was modified to avoid switching between retrieval and learning new information in the related question condition (see Fig. 1). In the guessing phase, 16 related questions and 16 target questions from the errorful learning condition were presented for 8 s, and participants were asked to generate their best guess. In the following learning phase, all 48 target questions were presented simultaneously with their correct responses for 5 s each. This created an average delay of 4.75 min between the guessing attempt and learning phases in both the related question and errorful learning conditions. After the learning phase, the final cued-recall test followed, which was the same as in the previous experiments.

### Results and discussion

During the learning phase, 125 answers to the target questions were correctly guessed in the errorful learning condition, which resulted in 8.8% of trials removed from subsequent analyses. The average number of correctly answered items per subject was 1.4 (*SD* = 1.2). Also, 247 responses to the related questions were correct, which constituted 17.3% of trials. As in Experiment 4, these trials were not removed from the analysis.

A one-way ANOVA on cued-recall performance (see Table 1 for descriptive statistics) revealed that performance differed across the three learning conditions, *F*(2, 176) = 5.48, *p* = .005, η_p_^2^ = .06. We further performed a series of Bonferroni-corrected paired-sample *t* tests to compare performance between those conditions. Performance in the errorful learning condition was significantly higher than in the related question condition, *t*(88) = 3.52, *p* = .002, *d* = 0.37, and in the short reading condition, *t*(88) = 2.48, *p* = .04, *d* = 0.26. There was no significant difference between the short reading and related question conditions, *t*(88) = 0.68, *p* = 1.00, *d* = 0.07.^7^

A Bayesian repeated-measures ANOVA yielded moderate evidence supporting a difference between the learning conditions, BF₁₀ = 5.13. Bayesian paired-samples *t* tests yielded strong evidence for an advantage of errorful learning over answering a related question, BF₁₀ = 32.80, while the evidence for a similar advantage over short reading was only anecdotal, BF₁₀ = 2.11. The comparison of short reading to answering a related question yielded moderate evidence in support of the null hypothesis, BF₀₁ = 6.84.

The results of Experiment 5 closely resembled those of Experiment 4. Despite the elimination of the requirement to switch between retrieval and learning of new information in the related question condition, performance in this condition was still indistinguishable from the short reading condition. Thus, we again failed to find any benefits of semantic activation triggered by guessing an answer to a related question. At the same time, we did replicate the benefits to learning accruing from attempting to guess an answer to the target question. Since such benefits arise only when the exact same question is the target of guessing attempts and learning, and are only observed when assessed against short reading times, it ultimately seems that they stem from additional time to process the target question itself.

## General discussion

Errorful learning has been recently proposed as an effective learning strategy that can be easily implemented in classroom settings (Clark & Bjork, 2014; Richland et al., 2009). When introducing a novel concept, instructors could first ask students a question concerning this concept, make them guess an answer, and not worry about any errors that those students may commit, assuming that their memory for this concept will improve even if their guesses are incorrect. But to make a strong claim that errorful learning is a strategy worth implementing in educational practice, one needs to prove its superiority over standard learning conditions. Thus, does errorful learning benefit memory over simply reading the same materials?

Previous studies that looked into this issue using trivia as learning materials found that errorful learning does indeed benefit memory compared with reading the study materials outright, but only when additional time to attempt a guess at the correct answer is provided (Kornell et al., 2009). Here, we replicated this pattern in all experiments that included this comparison (i.e., in Experiments 1, 4, and 5). But a more vital question is whether errorful learning still outstrips reading when the time devoted to learning across these strategies is equated. We systematically assessed this issue in the present study and found no evidence for the superiority of errorful learning of answers to trivia questions under equated learning time conditions in Experiment 1, a pattern which was also replicated in Experiment 2. We further assessed whether our inability to document the benefits of errorful learning reflects our particular choice of study materials, with benefits possibly limited to cases in which answers to trivia questions are sufficiently familiar to be easily linked to their respective questions. But in Experiment 3 we also failed to find evidence for any benefits of errorful learning for familiar answers. Altogether, the results of our investigation suggest that the benefits of errorful learning for trivia questions are elusive, arising when additional time is devoted to the guessing process but disappearing when the same amount of time is provided for the standard reading condition.

If differences in study time are so vital for the patterns of benefits of errorful learning, then one can ask how this time is used by the learners. One option is simply that enough time is necessary to first understand a question and then associate it with its answer. In our investigation, we used 5 s for study in the standard reading condition, extending it to 13 s when equating the reading and errorful learning conditions—the timings taken from a previous examination of the errorful learning of trivia questions and answers (Kornell et al., 2009). It is possible that the process of understanding complex materials such as trivia questions and associating correct answers to these questions is not complete within 5 s but is fully accomplished within 13 s. For the errorful learning condition, participants received 8 s to understand the question first, which meant that the remaining 5 s could be used solely for associating answers to these already processed questions.

Alternatively, we reasoned that there could be a specific process that takes place when a trivia question is read or guessed at. This would amount to the question activating a semantic network of plausible answers, possibly including the correct answer, with this activation facilitating encoding of the answer once the processing of a question is accomplished. This spreading semantic activation account of learning has been proposed as a mechanism of errorful learning when word pairs are studied (Grimaldi & Karpicke, 2012). We tested this account as applied to trivia questions in Experiments 4 and 5 by first asking participants questions related to the target questions, stipulating that these should also activate the common semantic network. However, asking those related questions failed to facilitate learning of the answers to target questions—both when these were presented immediately after their related question (Experiment 4) and when a delay was introduced between the related questions and their target questions (Experiment 5)—providing no evidence for the role of spreading semantic activation in this paradigm.

Notably, the lack of benefits for related questions in Experiment 5, which implemented a delay across related and target questions, is similar to results obtained with the pretesting paradigm (Carpenter et al., 2018), where a lecture is preceded with questions students do not know responses to. These studies have generally found that pretesting facilitates subsequent learning of answers to these questions during lecture but does not improve learning of other information contained in the same lecture (James & Storm, 2019; but see Sana & Carpenter, 2023). Thus, again, having more time to familiarize oneself with a question improves subsequent learning of the answer to this question, but such improved learning does not generalize beyond the queried information. We contend, thus, that in situations where questions are novel and their answers are not yet known to students, posing them before the correct answers are revealed is unlikely to lead to spreading semantic activation that would facilitate learning and instead serves to provide more time to comprehend those questions, a benefit that can be replicated with the reading strategy by simply extending study time.

At this stage we need to note that our results concerning the failure of answering a related question to promote memory for the target question and answer should be considered preliminary. In our study, we did not perform any a priori norming procedure to investigate how likely the initial questions were to activate the response pool corresponding to the target questions. Future studies should thus aim at measuring the relationship between the two questions to strengthen or refute the claims we made here.

One issue that merits additional discussion is the role of item-selection artifacts. Because errorful learning is defined as learning after formulating incorrect guesses, deriving the usual measure of performance in the errorful learning condition requires excluding those questions for which correct responses were formulated when the question was first asked. If these questions are not eliminated, then it is unclear to what extent subsequent memory performance reflects errorful learning or strengthening of already known answers via the mechanism of the testing effect (Roediger & Karpicke, 2006). However, as noted by Kornell et al. (2009), excluding correctly answered questions from the errorful learning condition puts this condition at a disadvantage compared with the reading condition. This is because correctly answered questions that are excluded from the errorful learning condition are necessarily the easiest questions that still contribute to correct responding in the reading condition.

Could our failure to document the benefits of errorful learning reflect merely a disadvantage of this strategy due to an item-selection artifact? We consider this possibility unlikely for two reasons. First, Kornell et al. (2009) addressed the issue of item-selection artifacts by examining errorful learning in the context of fictional trivia questions for which no correct answers exist. In this case, no exclusions were necessary, yet the patterns of results—which inspired the present investigation—were the same as for true questions: no benefits of errorful learning over reading with equated study time. Second, we also analyzed the results of all experiments without excluding questions correctly answered initially in the errorful learning condition (see Footnotes 2–6). Such analyses put the errorful learning condition at an advantage due to a small possible contribution of the testing effect to learning. Still, in none of the experiments were the results markedly different from those obtained when correctly answered questions were excluded. Thus, even without excluding any responses, errorful learning does not facilitate subsequent memory performance over and above reading.

Finally, one has to note that the present lack of benefits of errorful learning stands in stark contrast to research employing weakly related pairs of words, where clear benefits of incorrect guessing are observed even when study times are equated with the reading condition (e.g., Huelser & Metcalfe, 2012; Knight et al., 2012; Kornell et al., 2009). There is clearly a mechanism by which errorful learning does enhance memory even if it does not easily generalize to materials such as trivia. Given the prominent role of semantic activation as a mechanism to account for effects observed with weakly related word pairs (Grimaldi & Karpicke, 2012), one can speculate that such a mechanism does not apply to other types of materials. Related pairs of words chosen from associative norms are rich in preexisting semantic associations (Brainerd et al., 2008). Thus, this type of learning materials seems uniquely suited to revealing the role of spreading semantic activation that can result from guessing.

By contrast, materials such as trivia constitute a mix of elements of various levels of semantic transparency, ranging from completely novel arrangements, where nothing in the question points to the correct response, to examples of marginal knowledge (Berger et al., 1999; Cantor et al., 2015), where the semantic association is already established but is not strong enough to allow for correct responding without another presentation of the correct answer. It is possible that the effects of errorful learning depend on this strength of semantic relationship, with costs observed with no transparent relationship, as suggested by a trend in the present Experiment 3 for unfamiliar targets, or in the case of foreign language vocabulary (Butowska et al., 2022; Seabrooke et al., 2019), no effect for a relatively weak relationship, and an actual positive effect for marginal knowledge. This speculation awaits further research but nonetheless it is important to note that even if it were confirmed, it would relegate errorful learning to a status of an effective learning strategy for only a small minority of cases related to marginal knowledge that are likely specific to each learner and cannot be easily identified before the learning process commences. As it stands now, research underscoring the usefulness of errorful learning comes from studies employing materials that have very little in common with anything that students may wish to master. We thus believe that any demonstrations of the usefulness of errorful learning need to move beyond the pursuit of easily replicable but not easily generalizable effects observed for highly simplified study materials.

## Supplementary Information

Below is the link to the electronic supplementary material.

Supplementary file1 (DOCX 14.4 kb)

## Data Availability

All materials and data are available online (https://osf.io/hjefx). The study was not preregistered.

## Appendix

To make sure that the questions and answers could indeed be perceived by participants as related, after the completion of Experiments 4 and 5 we presented the materials created for this study to a new group of 39 participants, and had them reflect on the relatedness of the information queried by our questions. First, two questions from the related question condition were presented one above the other, and participants had to answer a question “To what extent do you think the answers to these questions might be related?” on a on a 1 (completely unrelated) to 7 (strongly related) scale. This was immediately followed by the presentation of the same questions but now together with their answers, and another question: “To what extent do you think the ACTUAL answers to these questions are related?”. To be able to check whether participants really perceived the expected and actual answers as related, half of the questions were presented with their related counterpart and the rest was repaired with other questions from the same set. There was a significant difference in relatedness scores between related (*M* = 4.68, *SD* = 0.26) and repaired questions (*M* = 1.69, *SD* = 0.59) both when presented on their own, *t*(38) = 12.42, *p* < .001, *d* = 1.99, and when presented with their answers (*M* = 4.38, *SD* = 0.61, and *M* = 1.47, *SD* = 0.21, respectively), *t*(38) = 12.2, *p* < .001, *d* = 1.95

**Table A1:** Cued recall performance as a function of pair relatedness, learning condition, and rated answer type in Experiments 4 and 5

	Pair relatedness					
	Least related			Most related		
Experiment and answer type	Errorful learning	Short reading	Related question	Errorful learning	Short reading	Related question
Experiment 4						
Expected	.63 (.06)	.56 (.05)	.52 (.06)	.60 (.06)	.55 (.07)	.55 (.06)
Actual	.62 (.06)	.53 (.05)	.54 (.06)	.67 (.06)	.59 (.08)	.59 (.06)
Experiment 5						
Expected	.66 (.06)	.55 (.05)	.55 (.05)	.55 (.07)	.53 (.07)	.54 (.07)
Actual	.64 (.05)	.54 (.05)	.56 (.06)	.62 (.07)	.60 (.07)	.59 (.07)

*Note.* Standard errors of the mean are given in parentheses

We then split the questions on the basis of those mean relatedness ratings, and thus created two sets of 16 questions with the highest relatedness ratings (*M* = 5.30, *SD* = 0.28 for the expected answers, and *M* = 5.03, *SD* = 0.31, for the actual answers) and two sets of 16 questions with the lowest relatedness ratings (*M* = 4.03, *SD* = 0.37 for the expected answers, and *M* = 3.73, *SD* = 0.38, for the actual answers). Because the target questions were used in all three learning conditions – errorful learning, related question, and short reading – we were able to compare cued-recall performance for these questions. This was done to ensure that the results of the main analyses were not obscured by any item-level effects stemming from differences in relatedness between question pairs. Cued-recall performance for these questions across the learning conditions is presented in Table A1. 

We first performed additional analyses only on the results for the 16 pairs of questions rated as having their expected answers most and least related. For Experiment 4, a 2 (relatedness: most related vs least related) x 3 (learning condition: errorful learning, related question, short reading) ANOVA was applied to final test performance. It produced only a significant main effect of learning condition, *F*(2,30) = 12.09, *p* < .001, *η*_*p*_^*2*^ = .45, which mirrored the findings from the main analysis, but the main effect of relatedness, *F*(1,15) = 0.002, *p* = .963, *η*_*p*_^*2*^ < .001, and the interaction, *F*(2,30) = 0.73, *p* = .491, *η*_*p*_^*2*^ = .05, were not significant. For Experiment 5, again the main effect of learning condition was significant, *F*(2,30) = 5.27, *p* = .011, *η*_*p*_^*2*^ = .26, while the main effect of relatedness was not, *F*(1,15) = 0.25, *p* = .625, *η*_*p*_^*2*^ = .02. This time, however, the interaction reached significance, *F*(2,30) = 3.79, *p* = .034, *η*_*p*_^*2*^ = .20. For the least related pairs there was a difference between the learning conditions, *F*(2,30) = 7.81, *p* = .002, *η*_*p*_^*2*^ = .34, with both the short reading and related questions condition resulting in lower performance than the errorful learning condition, *t*(15) = 3.44, *p* = .005, *d* = 0.49, and *t*(15) = 3.40, *p* = .006, *d* = 0.49, respectively, while not differing significantly from each other, *t*(15) = 0.40, *p* = 1.0, *d* = 0.006 (all *t*-tests are Bonferroni corrected). For the most related pairs, on the other hand, the conditions did not significantly differ from one another, *F*(2,30) = 0.18, *p* = .840, *η*_*p*_^*2*^ = .01.

The same ANOVA was then applied to final test performance for the 16 pairs of questions rated as having their actual answers most and least related. In Experiment 4, it again produced only a significant main effect of learning condition, *F*(2,30) = 10.15, *p* < .001, *η*_*p*_^*2*^ = .40, while the main effect of relatedness, *F*(1,15) = 0.43, *p* = .520, *η*_*p*_^*2*^ = .03, and the interaction, *F*(2,30) = 0.16, *p* = .856, *η*_*p*_^*2*^ = .01, were not significant. The same pattern was present for Experiment 5 data: a significant main effect of learning condition, *F*(2,30) = 4.16, *p* = .025, *η*_*p*_^*2*^ = .22, a nonsignificant main effect of relatedness, *F*(1,15) = 0.10, *p* = .752, *η*_*p*_^*2*^ = .01, and a nonsignificant interaction, *F*(2,30) = 2.56, *p* = .094, *η*_*p*_^*2*^ = .14.

Together, the results give further credence to our claim that preceding the to-be-learned information with a related question, aimed at targeting similar concepts, does not aid learning. In none of the analyses was there any inkling of a memory benefit in the related question condition compared to the short reading condition, even when only the most strongly related questions were considered. The only analysis that produced somewhat discrepant results was the one performed on the basis of the relatedness ratings for the expected answers in Experiment 5. Note, however, that even in this case the significant interaction was not caused by a memory boost in the related question condition. Instead, the benefit of errorful learning that was present across this study was eliminated there. We consider this finding to be an outlier, especially as it was absent from the analyses of Experiment 4 data which were based on the same relatedness ratings, as well as from the analyses of Experiment 5 data based on ratings for actual rather than expected answers. All in all, the analyses thus support the finding that the additional time spent on processing a related question is useless for aiding learning.

## Footnotes

1 Due to an error when setting up Experiment 1, later copied to all subsequent experiments, we failed to set an upper age limit for participating in our experiment. To mitigate any potential biasing effects on our results this might have caused, for each experiment we performed additional analyses on the restricted sample excluding participants aged over 60, which are reported in Supplemental Materials. The results were no different than those found in the full dataset.

2 A one-way ANOVA on the proportion of correct responses on the final test showed a significant difference between the learning conditions, *F*(2, 78) = 6.99, *p* = .002, η_p_^2^ = .15. The performance for errorful learning and long reading conditions was nearly identical and not significantly different (*M* = .84, *SD* = .18 and *M* = .83, *SD* = .16, respectively), *t*(39) = 0.63, *p* = 1.00, *d* = 0.10, but the performance for the short reading condition (*M* = .77, *SD* = .22) was significantly lower than in the errorful learning, *t*(39) = 3.24, *p* = .007, *d* = 0.51, or long reading condition, *t*(39) = 2.72, *p* = .029, *d* = 0.43.

3 For completion, we also report the results including the correctly answered items in the errorful learning condition. Here, we also found no significant difference between the errorful learning and reading (*M* = .80, *SD* = .18 and *M* = .79, *SD* = .17, respectively), *t*(70) = 0.73, *p* = .47, *d* = 0.09.

4 A repeated-measures ANOVA, with learning condition (errorful learning vs. long reading) and target familiarity (high vs. low), performed on the full dataset without excluding correctly answered questions, revealed a main effect of target familiarity, *F*(1, 40) = 204.8, *p* < .001, η_p_^2^ = .837. Overall, questions with familiar answers were answered correctly more often (*M* = .89, *SD* = .17) than questions with unfamiliar answers (*M* = .36, *SD* = .27). There was no significant main effect of learning condition, *F*(1, 40) = 1.36, *p* = .25 η_p_^2^ = .033. Also, the interaction was not significant, *F*(1, 40) = 3.22, *p* = .08, η_p_^2^ = .074. Planned comparisons showed no significant difference between the learning conditions neither for familiar answers (*M* = .90, *SD* = .15 for errorful learning and *M* = .88, *SD* = .18 for long reading), *t*(40) = 0.54, *p* = 1.00, *d* = 0.09, nor for unfamiliar answers (*M* = .39, *SD* = .27 for long reading and *M* = .34, *SD* = .27 for errorful learning), *t*(40) = 2.11, *p* = .23, *d* = 0.33.

5 Additional analyses for Experiments 4 and 5, which take relatedness ratings for related questions into account, can be found in the Appendix. To preview, the related question condition did not produce better memory performance compared with short reading even for questions with the highest relatedness ratings.

6 A one-way ANOVA on the proportion of correct responses on the final test including items correctly answered in the errorful learning condition showed a significant difference between the learning conditions, *F*(2, 178) = 12.08, *p* < .001, η_p_^2^ = .119. Performance in the errorful learning condition (*M* = .64, *SD* = .20) was significantly higher than in the related question condition (*M* = .56, *SD* = .22), *t*(89) = 4.41, *p* < .001, *d* = 0.47, and in the short reading condition (*M* = .57, *SD* = .22), *t*(89) = 3.88, *p* < .001, *d* = 0.41. There was no significant difference between the short reading and related question condition, *t*(89) = 0.78, *p* = 1.00, *d* = 0.08.

7 A one-way ANOVA on the proportion of correct responses on the final test including items correctly answered in the errorful learning condition showed a significant difference between the learning conditions, *F*(2, 176) = 13.57, *p* < .001, η_p_^2^ = .134. To unpick these differences, we performed a series of paired-sample *t* tests. Performance in the errorful learning condition (*M* = .64, S*D* = .22) was significantly higher than in the related question condition (*M* = .56, *SD* = .23), *t*(88) = 5.24, *p* < .001, *d* = 0.56, and in the short reading condition (*M* = .57, *SD* = .25), *t*(88) = 4.38, *p* < .001, *d* = 0.46. There was no significant difference between the short reading and related question conditions, *t*(88) = 0.68, *p* = 1.00, *d* = 0.07.

## Code availability

No code has been created for analytical purposes.
