# Supplementary material for: Errorful learning of trivia questions and answers: The role of study time
Source: Mem Cognit. 2024 Jul 25;53(3):804–19. doi: 10.3758/s13421-024-01608-6 (PMC12053351; doi:10.3758/s13421-024-01608-6)
Supplement: Supplementary file 1 — Supplementary file1 (DOCX 14.4 kb) [file 13421_2024_1608_MOESM1_ESM.docx]

**Supplemental Materials**

Analyses excluding participants aged 60 or over in Experiments 1-5.

**Experiment 1**

A one-way ANOVA showed a significant difference between the learning conditions, *F*(2,72) = 5.16, *p* = .008, η_p_^2^ = .13. Performance for the errorful learning and long reading conditions was nearly identical and not significantly different (*M* = .84, *SD* = .18, and *M* = .83, *SD* = .16, respectively), *t*(36) = 0.11, *p* = 1.00, *d* = 0.02. Performance for the short reading condition (*M* = .77, *SD* = .21) was, on the other hand, significantly lower than for the errorful learning condition, *t*(36) = 2.70, *p* = .032, *d* = 0.44, and the long reading condition, *t*(36) = 2.59, *p* = .042, *d* = 0.43.

**Experiment 2**

Performance in the errorful learning and long reading conditions was nearly identical and not significantly different (*M* = .78, *SD* = .18, and *M* = .78, *SD* = .17, respectively), *t*(66) = 0.08, *p* = .94, *d* = 0.01. A Bayesian paired-samples *t*-test comparing the errorful learning and long reading conditions yielded moderate evidence against any difference between study conditions: BF₀₁ = 7.44.

**Experiment 3**

A repeated-measures ANOVA with learning condition (errorful learning vs. long reading) and target familiarity (high vs. low) yielded a significant main effect of target familiarity, *F*(1,36) = 245.96, *p* < .001, η_p_^2^ = .87. Overall, questions with familiar answers were answered correctly more often (*M* = .88, *SD* = .18) than questions with unfamiliar answers (*M* = .33, *SD* = .25). There was no significant main effect of learning condition, *F*(1,36) = 3.83, *p* = .06 η_p_^2^ = .096. However, the interaction was significant, *F*(1,36) = 4.18, *p* = .048, η_p_^2^ = .104. We performed planned comparisons of the effectiveness of learning strategy separately for questions with familiar and unfamiliar answers. For familiar answers, there was no significant difference between the learning conditions (*M* = .88, *SD* = .17 for errorful learning and *M* = .87, *SD* = .19 for long reading), *t*(36) = 0.26, *p* = 1.00, *d* = 0.04. For unfamiliar answers, however, long reading was significantly better in supporting subsequent memory than errorful learning (*M* = .37, *SD* = .26, and *M* = .29, *SD* = .24, respectively), *t*(36) = 2.83, *p* = .037, *d* = 0.47.

**Experiment 4**

A one-way ANOVA revealed significant differences in final test performance between the learning conditions, *F*(2,168) = 6.23, *p* = .002, η_p_^2^ = .069. Performance in the errorful learning condition was significantly higher than in the related question condition (*M* = .63, *SD* = .20, and *M* = .57, *SD* = .22), *t*(84) = 3.16, *p* = .007, *d* = 0.34, and in the short reading condition (M = .57, SD = .22), *t*(84) = 2.82, *p* = .018, *d* = 0.31. There was no significant difference between the short reading and related question conditions *t*(84) = 0.35, *p* = 1.00, *d* = 0.04.

**Experiment 5**

A one-way ANOVA revealed that performance differed across the three learning conditions, *F*(2,148) = 4.17, *p* = .02, η_p_^2^ = .008. Performance in the errorful learning condition was significantly higher than in the related question condition (*M* = .61, *SD* = .24, and *M* = .56, *SD* = .23, respectively), *t*(74) = 2.58, *p* = .036, *d* = 0.30, and in the short reading condition (*M* = .56, *SD* = .26), *t*(74) = 2.74, *p* = .023, *d* = 0.32. There was no significant difference between the short reading and pre-question conditions, *t*(74) = 0.17, *p* = 1.00, *d* = 0.02.
